# Accurate classification of white blood cells by coupling pre-trained ResNet and DenseNet with SCAM mechanism

**DOI:** 10.1186/s12859-022-04824-6

**Published:** 2022-07-15

**Authors:** Hua Chen, Juan Liu, Chunbing Hua, Jing Feng, Baochuan Pang, Dehua Cao, Cheng Li

**Affiliations:** 1 Institute of Artificial Intelligence, School of Computer Science, Wuhan University, Wuhan, 430072 China; 2 Landing Artificial Intelligence Center for Pathological Diagnosis, Wuhan, 430072 China

**Keywords:** Deep learning, Spatial and channel attention, Transfer learning, Mixup, White blood cells classification

## Abstract

**Background:**

Via counting the different kinds of white blood cells (WBCs), a good quantitative description of a person’s health status is obtained, thus forming the critical aspects for the early treatment of several diseases. Thereby, correct classification of WBCs is crucial. Unfortunately, the manual microscopic evaluation is complicated, time-consuming, and subjective, so its statistical reliability becomes limited. Hence, the automatic and accurate identification of WBCs is of great benefit. However, the similarity between WBC samples and the imbalance and insufficiency of samples in the field of medical computer vision bring challenges to intelligent and accurate classification of WBCs. To tackle these challenges, this study proposes a deep learning framework by coupling the pre-trained ResNet and DenseNet with SCAM (spatial and channel attention module) for accurately classifying WBCs.

**Results:**

In the proposed network, ResNet and DenseNet enables information reusage and new information exploration, respectively, which are both important and compatible for learning good representations. Meanwhile, the SCAM module sequentially infers attention maps from two separate dimensions of space and channel to emphasize important information or suppress unnecessary information, further enhancing the representation power of our model for WBCs to overcome the limitation of sample similarity. Moreover, the data augmentation and transfer learning techniques are used to handle the data of imbalance and insufficiency. In addition, the mixup approach is adopted for modeling the vicinity relation across training samples of different categories to increase the generalizability of the model. By comparing with five representative networks on our developed LDWBC dataset and the publicly available LISC, BCCD, and Raabin WBC datasets, our model achieves the best overall performance. We also implement the occlusion testing by the gradient-weighted class activation mapping (Grad-CAM) algorithm to improve the interpretability of our model.

**Conclusion:**

The proposed method has great potential for application in intelligent and accurate classification of WBCs.

## Background

**Fig. 1:**
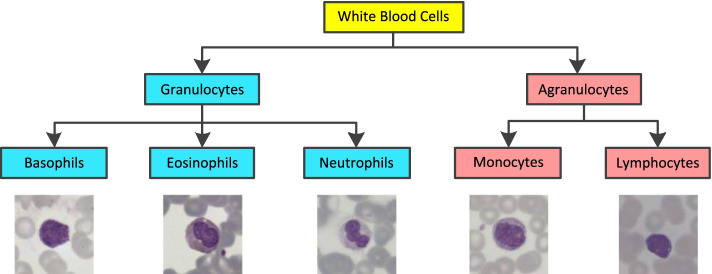
Examples of five types of WBC images

WBCs, also called leukocytes, are created in the bone marrow and lymphoid masses in the human immune system. These cells protect the human body from infections such as bacteria, viruses, and fungi [1–3]. Traditionally, WBCs are mainly divided into granulocytes and agranulocytes [4, 5]. The granulocytes contain basophils (0–1%), eosinophils (1–5%), and neutrophils (50–70%), while the agranulocytes include monocytes (2–10%) and lymphocytes (20–45%) [4, 6]. Figure 1 exhibits some examples of WBC images. If the number of WBCs in a human body is higher or lower than the reference values, which may lead to many kinds of diseases [7, 8]. Hence, to accurately classify different types of WBCs is necessary.

The classification technology of WBCs can be divided into three types: manual examination method, automated hematology analyzer detection method, and machine learning method. The manual examination method is considered the gold standard for discriminating WBCs [9, 10]. However, this approach is inefficient and its results rely on the experience and knowledge of the hematologists.

By comparison, the automated hematology analyzer detection method has the ability to address the above issues [11, 12]. The method is mainly based on different technologies, such as electrical impedance, radiofrequency conductivity, light scatter, fluorescent scatter, cytochemistry, etc. [13, 14], to automatically differentiate the WBC types, and can achieve high accuracy and efficiency. However, this method can not use the morphology of WBCs in blood smears for classification. Furthermore, it can not digitally preserve blood smears, so the retrospective study is not available. This means that once there is any abnormality in the detection device, hematologists have to re-collect blood smears and distinguish WBCs by manual examination.

Of late, the digital images of blood smears can be easily obtained due to the rapid development of digital microscope and information technology [15, 16]. Therefore, many computer-aided methods based on machine learning techniques including traditional machine learning based methods and deep learning based methods have been developed for automatically distinguishing different types of WBCs in blood cell images. The traditional machine learning based methods input the extracted discriminative features for representing WBCs into the classifier to implement the classification task. For instance, Alqudah et al. [17] investigated the feature extraction and classification of WBC based on using the combination of principal component analysis and three classifiers [probabilistic neural network, support vector machine (SVM), and random forest (RF)]. Duan et al. [18] extracted features such as texture, shape, and spectrum features from the segmented cells, and applied SVM to recognize the types of the WBCs. Sharma et al. [19] used the bio-inspired optimized grey wolf algorithm to find the optimal features, and then combined with SVM, decision tree, RF, and k-nearest neighbor classifiers to detect WBCs. Dong et al. [20] first extracted geometry, color, and texture features based on segmented WBCs, then used the feature selection algorithm based on classification and regression trees to remove irrelevant and redundant features, and finally analyzed the performance of the particle swarm optimization SVM. Although these classification approaches can yield good results, they highly rely on the selection of feature engineering. However, determining which features are selected for constructing a classification model is generally difficult.

Different from the traditional machine learning based methods, the deep learning based methods are able to automatically learn the features from images and simultaneously carry out classification. Thus, many deep learning based approaches have been developed and successfully applied to WBC classification. For instance, Ridoy et al. [21] verified the performance of the convolutional neural network (CNN)-based model they presented for automatically classifying WBCs on the BCCD (blood cell count and detection) dataset [22]. Mohamed et al. [23] proposed the deep learning + traditional learning hybrid framework for WBC classification. The deep learning is to yield the feature vector and the traditional machine learning is for WBC classification. They experimented several combinations on the BCCD dataset and found that the hybrid of a pre-trained 1.0 MobileNet-224 model and a logistic regression classifier reached the highest classification accuracy. In order to investigate the classification performance of different network structures, Habibzadeh et al. [24] transferred a variety of pre-trained Inception and ResNet models to the public BCCD dataset of WBCs and found that the 4-class classification results of fine-tuning all layers were better than those of just fine-tuning the last layers, and the ResNet models performed better than the Inception models. Kutlu et al. [25] obtained the similar results after experimenting various deep learning networks on the combination of the BCCD and the LISC (leukocyte images for segmentation and classifcation) datasets [26]. We think that the good performance of ResNet models may be attributed to the adoption of the skip connection mechanism, which creates a path propagating information from a lower layer directly to a higher layer, thus effectively alleviating the gradient vanishing problem and easing the model optimization. Recently, some fusion models have been proposed to improve the accuracy of classifying WBCs by combining several CNNs, e.g., CNN-RNN (recurrent neural network) [27], AlexNet-GoogleNet-DenseNet [28], etc. However, whether these models can inherit the advantages of each CNN needs to be further explored.

Nevertheless, the work of Chen et al. [29] has shown that ResNet and DenseNet respectively are good at reusing features and exploring new features, which helps to enhance the representation power of model. Based on their study, we develop a parallel CNN by combining ResNet and DenseNet modules to integrate the advantages of both. Besides, we add the SCAM attention module [30] to our network for adaptive feature refinement to further motivate the model to learn discriminative information from WBC images to address the problem of sample similarity. In addition, to deal with the imbalanced and insufficient data, data augmentation and transfer learning (TL) strategies are adopted in the training process of model. Meanwhile, the mixup method is used for modeling the vicinity relation between different kinds of training samples to improve the generalization ability of the proposed method. Finally, the Grad-CAM algorithm [31] is used for the occlusion testing to understand the decision-making process of the model.

The remainder of this paper is organized as follows: “Materials and methods” section introduces the data collection and processing and the proposed methods. “Experiments and results” section presents the experimental results and analysis. Finally, “Conclusion” section concludes this work.

## Materials and methods

### Data collection

We have collected four WBC datasets in this paper from several data sources. We intend to use these data to evaluate the performance of our method.

**Fig. 2:**
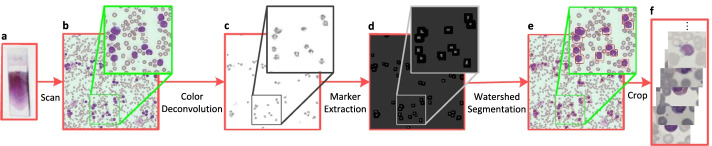
The process of WBC images generation. **a** Blood smear. **b** Microscopic image. **c** Color deconvolution to separate nucleus from background. **d** Marker extraction to locate WBCs. The white regions refer to the location of the nucleus. **e** Watershed algorithm to segment WBCs. **f** Crop to extract WBC images

From our cooperative medical institutions, we acquired 150 blood samples from 150 subjects. All samples are anonymized, so there is no concern about privacy. These samples were smeared, stained with Wright-Gimsa [32, 33], and scanned by the micro-scanning imaging device with high resolution to obtain the digital images. For each image, the WBC images with the size of 1280 $\times$ 1280 pixels were extracted by utilizing our own developed cell segmentation method. Our approach consists of color deconvolution [34], marker extraction, and watershed algorithm [35]. Marker extraction is to locate nucleus and then locate cells. The specific process of locating nucleus includes image binarization, hole filling, morphology opening operation, dilate operation, distance transformation, and morphology reconstruction. Figure 2 illustrates the generation process of WBC images. All images were definitively labeled by the team of hematologists. Consequently, we collected 22645 WBC images, including 224 basophils, 968 monocytes, 539 eosinophils, 10469 neutrophils, and 10445 lymphocytes.

Considering that the quantity and diversity of data is of great importance for training a model with excellent performance [36], this study releases the largest freely available WBC image dataset (called the LDWBC dataset) we have known so far to help facilitate the development of clinical hematology.

From LISC database, we obtained 242 WBC images. The size of each WBC image is 720 $\times$ 576 pixels. All the images were manually segmented and classified into five types by hematologists, consisting of 53 basophils, 48 monocytes, 39 eosinophils, 50 neutrophils, and 52 lymphocytes.

From BCCD database, we collected 12444 WBC images, which were divided into four categories: 3098 monocytes, 3120 eosinophils, 3123 neutrophils, and 3103 lymphocytes. The images in the dataset were cropped images of size 320 $\times$ 240 pixels.

From Raabin database [37], we downloaded 14514 WBC images, comprising 301 basophils, 795 monocytes, 1066 eosinophils, 8891 neutrophils, and 3461 lymphocytes at resolutions of 575 $\times$ 575.

Table 1 summarizes the four publicly available WBC datasets. It is noticed that the images in the LISC and BCCD datasets have low signal-to-noise ratio due to the inclusion of a large number of irrelevant background elements, which may have a negative impact on the performance of the model. Thereby, we cropped the WBC images in the LISC dataset based on the provided mask images of WBC. Meanwhile, we also extracted WBC images from the BCCD dataset by using our cell segmentation method. A total of 12336 W

**Table 1:** The image information in the four datasets

Dataset	Image number					Total	Pixel size
	B	M	E	N	L		
LDWBC	224	968	539	10469	10445	22645	1280 $\times$ 1280
LISC	53	48	39	50	52	242	720 $\times$ 576
BCCD	–	3098	3120	3123	3103	12444	320 $\times$ 240
Raabin	301	795	1066	8891	3461	14514	575 $\times$ 575

*B* basophi, * M* monocyte, * E* eosinophi, * N* neutrophil, * L* lymphocyte

BC images were obtained, and another 108 images were excluded from this study since they did not contain WBC or contained only a small fraction of WBC. As a note, most of WBCs are located at the edges of the images in the BCCD dataset so the cropped WBC images still contain a lot of noise.

### Classification model

**Fig. 3:**
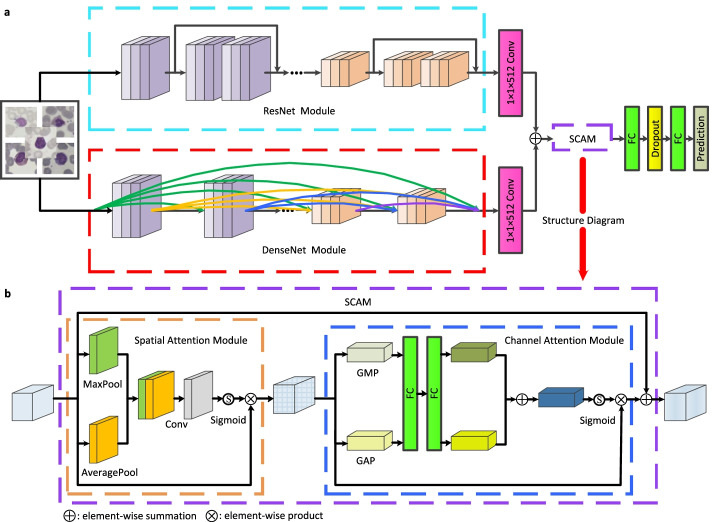
**a.** The architecture of our model. **b.** The structure diagram of the SCAM block used in **a**. Conv: convolutional; FC: fully-connected; GAP: global average pooling; GMP: global max pooling

Figure 3a depicts the architecture of our model. In the parallel network, ResNet and DenseNet are selected to share their respective advantages: the former encourages the features reuse while the latter is able to explore new features, which are both significant for learning good representations. To fuse their extracted features, we respectively selected the middle layers and removed the last fully-connected (FC) layers of them (named ResNet and DenseNet modules), and then we used a convolutional layer (kernel size: 1 $\times$ 1, number of filters: 512, size step: 1) to adjust the number of channels of the feature maps output by these two modules to ensure that the feature maps have the same size. Given the important role of attention in human perception, i.e., humans do not attempt to handle the whole scene but selectively concentrate on the prominent parts to better capture the visual structure [38]. Inspired by this, since the nucleus of WBC contains a large amount of discriminative information about the cell, we implanted a self-attention module into the model to improve the representation power of our network for the nucleus and thus overcome the limitation of sample similarity. The SCAM block shown in Fig. 3b is adopted, with the aim that the module includes both the spatial attention module (SAM) and channel attention module (CAM), where the SAM emphasizes where the important features are while CAM emphasizes what are the meaningful features in the feature maps. Finally, we sequentially stacked two FC layers to perform our WBC classification task. To alleviate the overfitting of the model, the dropout method was used before the last FC layer.

Although CNNs are highly effective in many applications, especially in image classification, training CNNs with high accuracy usually relies on massive data to help them understand the underlying patterns of data [39, 40]. Unfortunately, building large-scale WBC image data is extremely difficult clinically since the collection and annotation of WBC data are complex and expensive. However, TL relaxes the assumption that the training and test data must be independent and identically distributed [39], which means that it can use the knowledge learned from a similar domain to tackle a given domain task thus addressing the problem of limited data in the target domain. Some recent studies have fruitfully exploited TL in fields such as biomedicine [41–43], motivating us to also utilize TL to deal with insufficient WBC data. In addition, the low-level features extracted by CNNs are standard and regardless of the dataset utilized while the top-level features extracted are abstract and heavily rely on the dataset and task selected [44]. However, ResNet50 [45] and DenseNet121 [46] pre-trained on the ImageNet dataset have learned enough low-level features such as color, geometry, texture, etc., and features similar to these are also present in WBC images. Also based on this consideration, we implanted the parameters of the middle layers of these two pre-trained models into our model to enable our network to better concentrate on learning top-level features from WBC images to accomplish our classification task.

### Data processing

#### Data augmentation

**Fig. 4:**
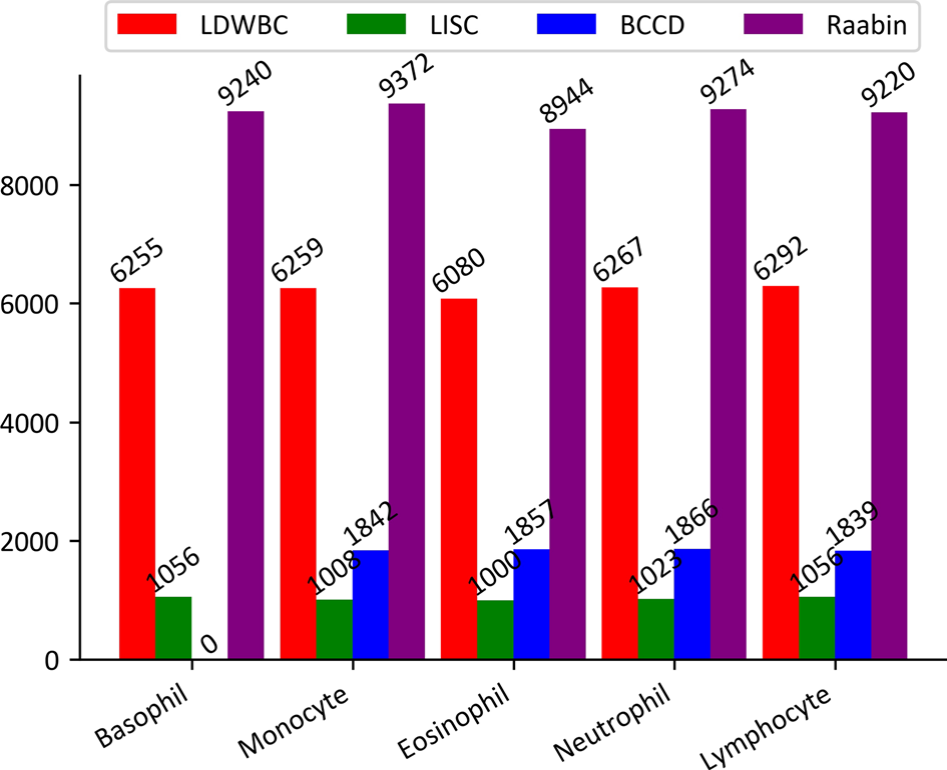
Distribution for each category in the four augmented training sets

Despite applying TL method to deep learning model can effectively address the issue of insufficient WBC data to a certain extent, deep learning model is also generally very sensitive to category imbalance [47]. However, there is a natural imbalance in the number of each type of WBCs in the human body. Hence, to tackle this problem, the data augmentation strategies are employed [48]. Meanwhile, data augmentation also increases the amount of training data, improving the generalization ability of model. In this work, for the LDWBC, LISC, and Raabin datasets, data augmentation was respectively performed on the training sets by randomly combining several transformation operations including rotation, flipping, translation, etc. Noted that, for the BCCD dataset, the training set has been augmented. For the four datasets, the number of images in each augmented training set is displayed in Fig. 4.

On the basis of the recommended computational requirements of ResNet model or DenseNet model, the uniform size of 224 $\times$ 224 dimension for all WBC images in these four datasets needs to be established. Then, we randomly split the LDWBC and LISC datasets into training, validation, and test sets respectively in a 3:1:1 ratio. Considering that the BCCD and Raabin datasets have included test sets, we randomly divided the training data in these two datasets into training and validation sets respectively with a ratio of 3:1. The training set is used to fit and update the model parameters, the validation set is for model selection and parameter adjustment, and the test set aims to objectively assess the performance of the trained model. Table 2 presents the number of WBC images for different sets.

**Table 2:** The number of images in different sets in the four datasets

Dataset	Total number	Training set	Validation set	Test set
LDWBC	22645	13587	4529	4529
LISC	242	145	48	49
BCCD	12336	7404	2467	2465
Raabin	14514	7631	2544	4339

#### Mixup operation

Data augmentation assumes that the samples in the vicinity share the same category while ignoring the vicinity relation between samples of different categories. However, the study of Zhang et al. [49] has demonstrated that the mixup method models this vicinity relation by training the model on convex combinations of paired samples and their labels, acting as a regularizer to suppress overfitting of the model. Inspired by their work, we combine data augmentation and mixup operation for the training data to further improve the generalization of the model.

**Fig. 5:**
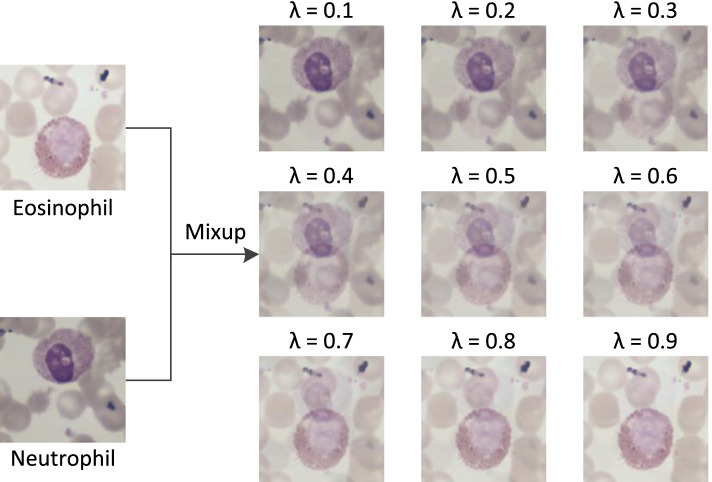
An example of the mixup operation for constructing a virtual training sample

The details of the mixup operation are as follows: Suppose $(x_{u}, y_{u})$ and $(x_{v}, y_{v})$ are two samples randomly selected from the training data, where $x_{u}$ and $x_{v}$ denote the pixel matrix respectively, and $y_{u}$ and $y_{v}$ refer to the corresponding label, represented by one-hot encoding. The virtual instance (*x*, *y*) is constructed by mixup operation:

1 $$\begin{aligned} x= & {} \lambda *x_{u}+(1-\lambda )*x_{v} \end{aligned}$$

2 $$\begin{aligned} y= & {} \lambda *y_{u}+(1-\lambda )*y_{v} \end{aligned}$$

where $\lambda$ $\in$ [0, 1] represents the weight factor that satisfies the distribution of Beta ($\alpha$, $\alpha$) and $\alpha$ $\in$ (0, +$\infty$) is one parameter. To help understand the generation of virtual samples via mixup operation, an example is provided in Fig. 5.

### Model training

All the models were trained, validated, and tested on a 64-bit ubuntu 16.04 operating system with Intel E5-2650 v4 2.20 GHz CPU, 256 Gb RAM, NVIDIA TITAN Xp 12 Gb GPU. For training, the RAdam optimizer [50] is utilized to minimize the categorical cross-entropy loss in Eq. (3). The parameter configuration is revealed in Table 3.

3 $$\begin{aligned} loss = -[ylog({\hat{y}})+(1-y)log(1-{\hat{y}})] \end{aligned}$$

where y and ${\hat{y}}$ respectively denote true label and predicted label.

**Table 3:** The parameter configuration of models

Parameters	Value
Dropout ratio	0.5
Initial learning rate	0.00001
Batch size	16
Epoch	100

## Experiments and results

We started by evaluating the impact of the mixup operation on model performance. The effects of several different attention methods were then compared. After that, the contribution of the ResNet and DenseNet modules and the attention module in our model, and the effort of TL for the model were verified by ablation studies. Then, the proposed model was compared with five representative networks on the four WBC datasets. We finally applied the Grad-CAM algorithm for the occlusion testing to help explain the decision-making process of our model.

### Performance metrics

The overall accuracy (OA), average precision (AP), average recall (AR), and average F1-score (AF1) are utilized to evaluate the ability of the model to identify WBC images. OA is calculated by dividing the number of correctly classified samples by the total number of samples. The other three evaluation criteria are stated as:

4 $$\begin{aligned} AP= & {} \frac{1}{N}\sum \limits _{c = 0}^{N - 1} {\frac{{TP(c)}}{{TP(c) + FP(c)}}} \end{aligned}$$

5 $$\begin{aligned} AR= & {} \frac{1}{N}\sum \limits _{c = 0}^{N - 1} {\frac{{TP(c)}}{{TP(c) + FN(c)}}} \end{aligned}$$

6 $$\begin{aligned} AF1= & {} \frac{{2*AP*AR}}{{AP + AR}} \end{aligned}$$

where *N* is the number of classes, and *c* represents that a class takes it as positive class and the other classes as negative class. *TP* (true positive): number of correctly classified positive samples; *FP* (false positive): number of misclassified negative samples; *TN* (true negative): number of correctly classified negative samples; *FN* (false negative): number of misclassified positive samples.

### Investigation on effect of mixup operation on model

According to Eqs. (1) and (2), the degree of linear interpolations of training samples depends on the value of the parameter $\alpha$. Therefore, we assessed the effect of setting the parameter between 0 and 1 with step 0.2 on the classification performance of our model. Table 4 displays the classification results of our model on the LDWBC test set. It can be seen from this table that the model trained with the virtual samples can yield higher scores than that trained with the raw samples ($\alpha = 0$). And, we also find that our model acquires the best performance when $\alpha = 0.2$. So, the value of $\alpha$ is set to 0.2 for generating the virtual training samples to construct our model.

**Table 4:** The classification results (%) under different $\alpha$ settings on our LDWBC test set

$\alpha$	OA	AP	AR	AF1
0	97.42	**93**.**51**	92.35	92.66
0.2	**97**.**84**	91.61	**96**.**38**	**93**.**82**
0.4	97.70	90.48	95.64	92.92
0.6	97.79	91.07	94.51	92.56
0.8	97.42	90.58	92.75	91.40
1.0	97.79	91.97	94.04	92.86

Best results are in bold

**Fig. 6:**
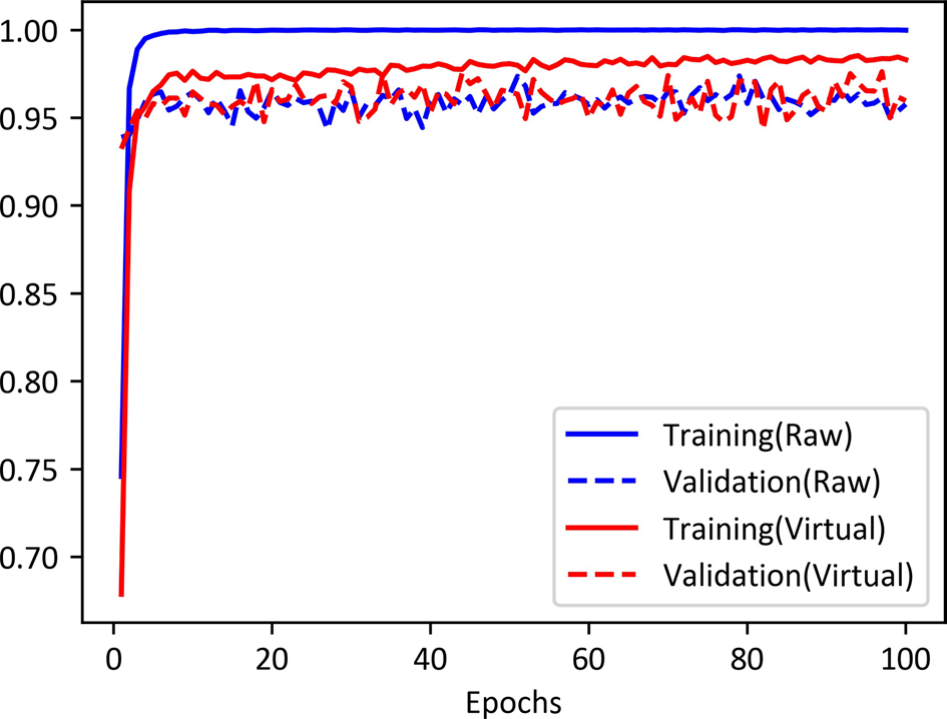
Effect of mixup operation on train and validation sets

We also respectively plotted the curves ($\alpha = 0$ and $\alpha = 0.2$) of the training and validation accuracies along with training epochs in Fig. 6, which shows that the model trained with the raw data is overfitting. The accuracy on the training set reaches 100% after several epochs, whereas the highest accuracy on the validation set is only 97.37%. On the contrary, the training and validation accuracies of the model trained with the virtual data are very close (98.53% and 97.62%), which illustrates that using the virtual samples instead of the raw ones can get more robust models. After using virtual data, although the accuracy rate on the validation set has some fluctuations, it has been improved to a certain extent. In addition, since the accuracy of the training set without using virtual data has approached 100%, the update of the network has become slow. We considered that the network has fallen into a stopping process at this time, so the accuracy of the validation set has not changed much, which seems more stable.

### Comparison of different attention methods

**Fig. 7:**
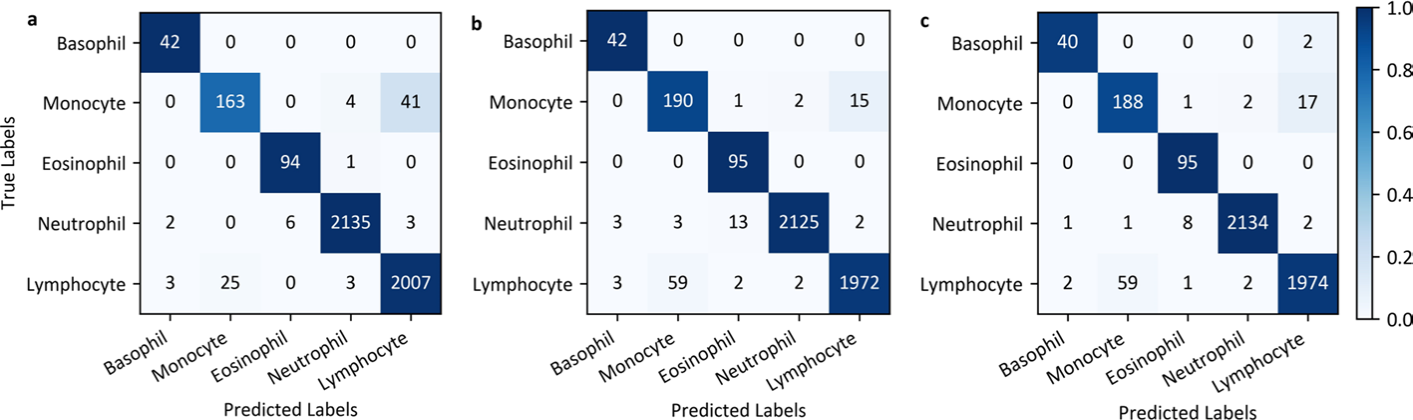
The confusion matrices for classification on our LDWBC test set. **a** CAM. **b** CSAM. **c** SCAM

Table 5 lists the effects of several common attention modules and their arrangement methods on the performance of model. From this table, it can be found that whether using channel attention or spatial attention or their combination can enhance the representation ability of network. However, we also find that the model seems to perform better when utilizing only channel attention. For further insight into the classification results, Table 6 exhibits the accuracy of model in identifying different types of WBCs. We can see that compared to using only channel or spatial attention, the parallel arrangement (CAM // SAM) does not improve the performance of model while the sequential arrangement (CSAM and SCAM) significantly raise the ability of model to recognize monocytes. This shows that the attention maps generated by the latter are finer than those generated by the former. To reveal the classification effect of the model using CAM, CSAM, and SCAM in more detail, Fig. 7 provides the corresponding confusion matrices. From Fig. 7 we can clearly see that the model performs best on lymphocytes but worst on monocytes by using CAM. In contrast, the model used CSAM or SCAM performs more balanced on these two types of WBCs. This indicates that the spatial attention method indeed enhances the representation ability of model to the nucleus. Finally, the further comparison shows that SCAM performs more balanced on all categories of WBCs compared to CSAM. This is due to the fact that CAM and SAM have different functions and therefore the order of combination impacts the performance of model.

**Table 5:** The performances (%) of models with different attention methods on our LDWBC test set

Attention method		OA	AP	AR	AF1
No		97.55	92.15	93.36	92.61
CD	SE [51, 52]	97.75	91.87	96.21	93.92
	ECA [53]	97.37	90.29	93.05	91.24
	CAM [30]	**98**.**06**	**93**.**51**	95.06	**94**.**17**
SD	SAM [30]	97.75	91.78	95.49	93.56
TD	CAM // SAM [30]	97.73	91.73	94.27	92.90
	CAM + SAM (CSAM) [30]	97.68	89.49	**97**.**43**	93.11
	SAM + CAM (SCAM) [30]	97.84	91.61	96.38	93.82

Best results are in bold; *CD* channel dimension, *SD* spatial dimension, *TD* two dimensions, // parallel, + sequential

**Table 6:** The accuracies (%) of models with different attention methods for each category on our LDWBC test set

Attention method		B	M	E	N	L
No		97.62	71.63	**100**	99.30	98.23
CD	SE [51, 52]	**100**	85.58	98.95	**99**.**67**	96.86
	ECA [53]	**100**	67.79	**100**	99.49	97.99
	CAM [30]	**100**	78.37	98.95	99.49	**98**.**48**
SD	SAM [30]	97.62	84.13	98.95	99.30	97.45
TD	CAM // SAM [30]	97.62	77.40	98.95	99.39	97.99
	CAM + SAM (CSAM) [30]	**100**	**91**.**35**	**100**	99.02	96.76
	SAM + CAM (SCAM) [30]	95.24	90.38	**100**	99.44	96.86

Best results are in bold;* CD* channel dimension, * SD* spatial dimension, * TD* two dimensions, // parallel, + sequential, * B* basophil, * M* monocyte, * E* eosinophil, * N* neutrophi, * L* lymphocyte

### Ablation study on model

Since we have evaluated the role of SCAM module in our model in the previous section, here we only assessed the contribution of the ResNet and DenseNet modules to the model by performing an ablation study. Table 7 lists the comparison results on different performance metrics. It can be seen from this table that the performance of the model decreases regardless of which branch is removed from the model, which shows that the advantages of the ResNet and DenseNet modules are compatible, enhancing the ability of our model to exploit the information in WBC images.

**Table 7:** The classification results (%) of the proposed components on our LDWBC test set

Our model			OA	AP	AR	AF1
RM	DM	SCAM				
	$\checkmark$	$\checkmark$	97.11	89.69	92.88	91.15
$\checkmark$		$\checkmark$	97.75	90.52	94.45	92.25
$\checkmark$	$\checkmark$	$\checkmark$	**97**.**84**	**91**.**61**	**96**.**38**	**93**.**82**

Best results are in bold;* RM* ResNet module, *DM* DenseNet module

Further, the effect of TL method on our model was also validated via ablation study. Tables 8 and  9 show the overall classification results of the model and the classification accuracy for each category, respectively. As can be seen from Tables 8 and  9, the use of TL method in any branch significantly enhances the ability of the model to identify basophils and monocytes. And the simultaneous use of TL method in both branches further effectively raises the classification ability of model on monocytes. This implies that TL enables the model to better learn the abstract features in WBC images and thus improves the representation ability of model. This also shows that TL in WBC classification task is an effective method for the limited training data.

**Table 8:** The classification results (%) of the TL method on our LDWBC test set

Our model					OA	AP	AR	AF1
RM		DM						
TL	No TL	TL		No TL				
$\checkmark$				$\checkmark$	**97**.**84**	91.30	95.35	93.21
	$\checkmark$	$\checkmark$			97.46	92.30	93.04	92.45
	$\checkmark$			$\checkmark$	96.20	**92**.**34**	82.64	85.88
$\checkmark$		$\checkmark$			**97**.**84**	91.61	**96**.**38**	**93**.**82**

Best results are in bold;* RM* ResNet module,* DM* DenseNet module

**Table 9:** The accuracies (%) of the TL method for each category on our LDWBC test set

Our model					B	M	E	N	L
RM		DM							
TL	No TL	TL		No TL					
$\checkmark$				$\checkmark$	95.24	84.62	**100**	99.11	97.79
	$\checkmark$	$\checkmark$			**100**	69.71	97.89	99.25	98.33
	$\checkmark$			$\checkmark$	78.57	39.42	96.84	**99**.**49**	**98**.**87**
$\checkmark$		$\checkmark$			95.24	**90**.**38**	**100**	99.44	96.86

Best results are in bold;* RM* ResNet module, * DM* DenseNet module, * B* basophil, * M* monocyte, * E* eosinophil, * N* neutrophil, * L* lymphocyte

### Comparison with other methods

**Fig. 8:**
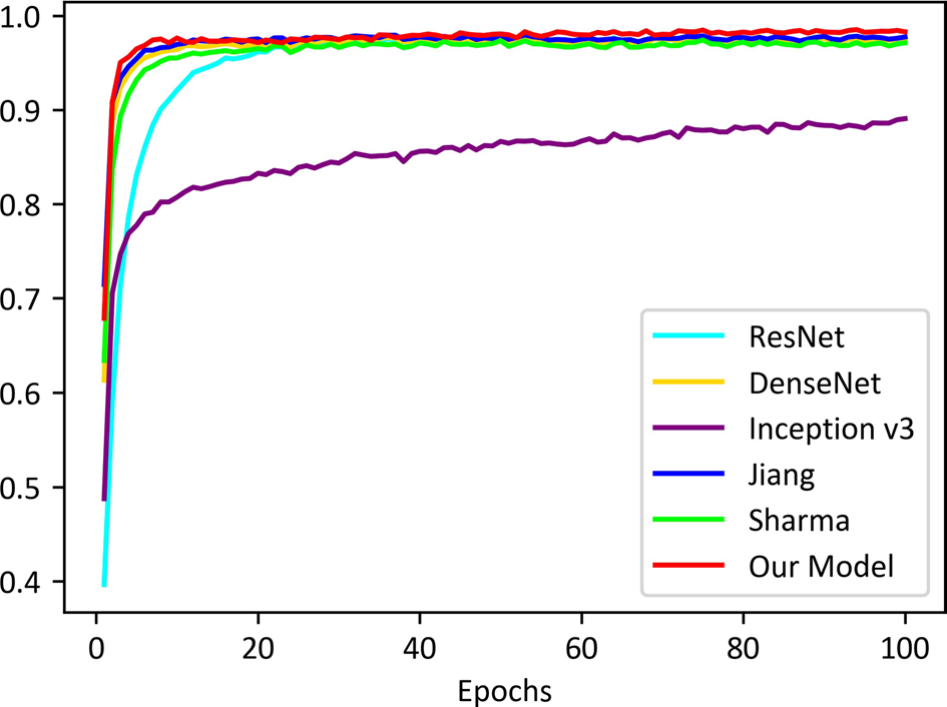
Train accuracy of all models on our LDWBC dataset

**Fig. 9:**
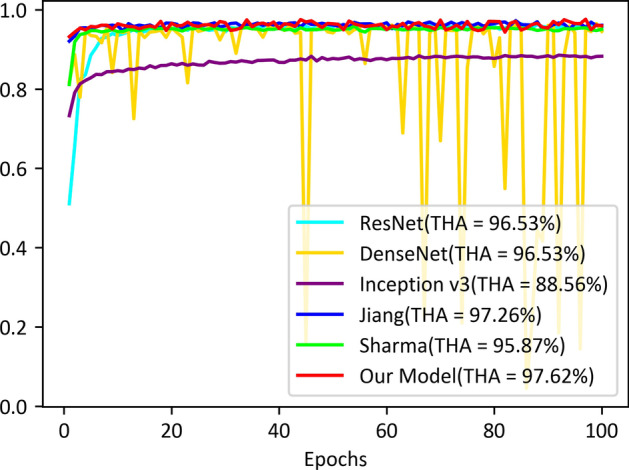
Validation accuracy of all models on our LDWBC dataset. THA refers to the highest accuracy

To evaluate the classification performance, we compared our model with five state-of-the-art methods on the four WBC datasets. All methods have the same parameter configuration. The models were trained on the training sets both on raw data and virtual data for the LDWBC dataset, and the one with the highest accuracy on the validation set for each method was selected as the final model. We evaluated the final models on the test sets, and the comparison results are shown in Table 10. As can be seen from Table 10, the performances of most models are improved by using mixup operation. Meanwhile, our model yields the best classification results. Moreover, we also compared the details of the training process of the proposed model with the five models on the LDWBC dataset, and the results are shown in Figs. 8 and 9 respectively. As can be seen from the figures, not only does our model obtains the highest accuracies in both training and validation sets, but also its performance fluctuates very slightly along the epochs of training. The results once again demonstrate that our model is robust and has strong adaptability for data. In addition, the performances of these models based on mixup operation were also compared on the other three datasets (See Table 11). In Table 11, the performance of our model ranks first on the BCCD and Raabin datasets and tied for second on the LISC dataset. These results collectively demonstrate that our model has excellent overall classification performance.

**Table 10:** The comparing results (%) of different methods for raw data and virtual data on our LDWBC test set

Model	Training set	OA	AP	AR	AF1
ResNet [45]	Raw	96.47	89.24	87.71	88.43
	Virtual	96.60	86.29	91.35	88.63
DenseNet [46]	Raw	96.53	89.07	90.28	89.41
	Virtual	96.82	88.09	93.25	90.49
Inception v3 [54]	Raw	89.40	70.57	68.17	69.06
	Virtual	88.67	73.27	63.59	67.23
Jiang [55]	Raw	97.11	90.85	90.68	90.75
	Virtual	97.37	91.08	92.31	91.61
Sharma [56]	Raw	95.72	85.62	82.08	83.16
	Virtual	95.78	89.01	79.84	83.46
Our model	Raw	97.42	**93**.**51**	92.35	92.66
	Virtual	**97**.**84**	91.61	**96**.**38**	**93**.**82**

Best results are in bold

**Table 11:** The comparing results (%) of different methods on the LISC, BCCD, and Raabin test sets

Dataset	Model	OA	AP	AR	AF1
LISC	ResNet [45]	93.88	95.48	92.50	92.98
	DenseNet [46]	**97**.**96**	98.33	**97**.**50**	97.80
	Inception v3 [54]	75.51	74.88	74.57	73.74
	Jiang [55]	**97**.**96**	**98**.**46**	**97**.**50**	**97**.**87**
	Sharma [56]	95.92	96.79	95.00	95.47
	Our model	**97**.**96**	98.33	**97**.**50**	97.80
BCCD	ResNet [45]	84.71	87.06	84.73	85.15
	DenseNet [46]	87.14	89.36	87.16	87.48
	Inception v3 [54]	62.80	67.71	62.79	63.52
	Jiang [55]	86.77	89.28	86.79	87.10
	Sharma [56]	87.02	89.15	87.03	87.31
	Our model	**88**.**44**	**90**.**84**	**88**.**45**	**88**.**73**
Raabin	ResNet [45]	96.36	92.87	96.15	94.28
	DenseNet [46]	97.12	94.02	97.07	95.42
	Inception v3 [54]	89.56	78.39	88.31	82.47
	Jiang [55]	96.13	91.69	97.00	93.97
	Sharma [56]	95.99	92.62	95.08	93.50
	Our model	**98**.**71**	**97**.**18**	**98**.**42**	**97**.**78**

Best results are in bold

We also present the classification accuracy of all models on these four datasets for each category of WBCs in Table 12. We find that our method displays excellent performance for almost all types of WBC on each dataset compared to other methods, especially on monocytes, which again shows the promising performance of our method. We also find that almost all methods are able to identify each type of WBC well on the LISC and Raabin datasets. However, all methods perform worse on the BCCD dataset than on the other datasets, which is likely attributable to the cropped WBC images in the dataset still having a low signal-to-noise ratio.

**Table 12:** The accuracies (%) of models for each category on the test sets of the four datasets

Dataset	Model	B	M	E	N	L
LDWBC	ResNet [45]	80.95	83.17	97.89	98.51	96.22
	DenseNet [46]	90.48	81.73	98.95	99.21	95.88
	Inception v3 [54]	42.86	37.50	52.63	93.48	91.46
	Jiang [55]	90.48	74.04	**100**	**99.44**	97.60
	Sharma [56]	64.29	50.48	87.37	99.21	**97.84**
	Our model	**95.24**	**90.38**	**100**	**99.44**	96.86
LISC	ResNet [45]	**100**	62.50	**100**	**100**	**100**
	DenseNet [46]	**100**	**87.50**	**100**	**100**	**100**
	Inception v3 [54]	91.67	50.00	63.64	85.71	81.82
	Jiang [55]	**100**	**87.50**	**100**	**100**	**100**
	Sharma [56]	**100**	75.00	**100**	**100**	**100**
	Our model	**100**	**87.50**	**100**	**100**	**100**
BCCD	ResNet [45]	–	**75.00**	79.55	84.38	**100**
	DenseNet [46]	–	**75.00**	84.42	89.21	**100**
	Inception v3 [54]	–	51.95	65.58	69.24	64.38
	Jiang [55]	–	72.73	84.58	89.86	**100**
	Sharma [56]	–	73.86	**85.23**	89.21	99.84
	Our model	–	74.84	**85.23**	**93.72**	**100**
Raabin	ResNet [45]	**100**	88.03	98.14	95.83	98.74
	DenseNet [46]	**100**	92.31	97.83	96.88	98.36
	Inception v3 [54]	92.13	81.62	86.96	90.00	90.81
	Jiang [55]	**100**	92.74	98.76	95.08	98.45
	Sharma [56]	**100**	85.04	96.27	95.94	98.16
	Our model	**100**	**94.87**	**99.07**	**98.65**	**99.52**

Best results are in bold;* B* basophil, *M* monocyte, *E* eosinophil, *N* neutrophil, *L* lymphocyte

### Interpretability of model

**Fig. 10:**
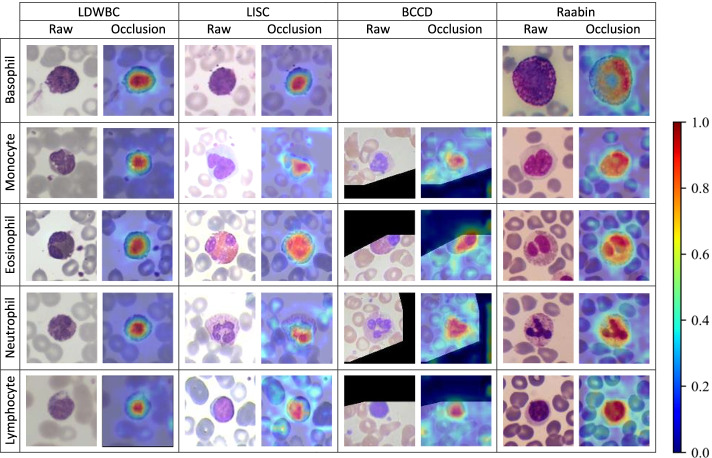
Several visualization examples are selected from the test sets of the four datasets. For each set, the left column is the raw input image, and the right column is the occlusion map generated by superimposing heatmap on the raw input image

In order to investigate the interpretability of our model, the occlusion testing was performed via utilizing the Grad-CAM algorithm to visualize the regions which had the greatest impact on model decisions. In our model, the output of the SCAM module was made transparent to the prediction of each type of WBC image, as shown in Fig. 10. In Fig. 10, the red regions on the occlusion map represent the areas where the model pays the most attention during the classification, while the blue regions receive the least attention, which can be decoded by the color bar on the right. We find that the salient areas of the occlusion maps are located on the nucleus, which indicates that the model uses features extracted from specific regions in the input WBC images and draws corresponding classification conclusions.

## Conclusion

In the present study, a novel deep learning method is developed to automatically and accurately differentiate WBCs. Our proposed method is able to learn better feature representation by integrating the advantages of ResNet and DenseNet. Moreover, the method also benefits from the guidance of the SCAM mechanism, further enhancing the representation ability of the model via emphasizing the meaningful features in WBC images in two independent dimensions of space and channel, which helps to tackle the issue of sample similarity. Since spatial attention and channel attention have different functions, different arrangement methods will yield different classification results. Considering that the imbalanced or insufficient training data may negatively affect the performance of the deep learning model, we adopt data augmentation and TL methods respectively. Furthermore, we use mixup method in addition to the dropout technique to model the vicinity relation between training samples of different classes to form a strong regularizer to further improve the generalization ability of the model. On the four WBC datasets, our method not only achieves superior overall classification performance but also performs well on each class of WBCs compared to other state-of-the-art methods. Finally, the occlusion testing is implemented using the Grad-CAM algorithm to visualize the discriminative areas of our model, thereby improving the explainability of the classification results.

Although the results of our method are promising, there also exist several limitations. We should improve the loss function to decrease the intra-class variations and increase the inter-class differences simultaneously to further raise the representation power of our method as part of future work. This is because the cross-entropy loss function penalizes the misclassified samples to separate the features of different categories, but ignores the differences between these samples. Apart from this, the current classification is based on five major subtypes of WBCs. However, more subtype classification is also a challenge for future study.

## Data Availability

The four WBC datasets analysed during the current study are publicly available through the following links: LDWBC: http://ldwbc.biodwhu.cn/LDWBC/ LISC: http://users.cecs.anu.edu.au/%7Ehrezatofighi/Data/Leukocyte%20Data.htm BCCD: https://www.kaggle.com/datasets/paultimothymooney/blood-cells Raabin: https://raabindata.com/free-data/

## Abbreviations

WBC: White blood cell
SCAM: Spatial and channel attention module
TL: Transfer learning
LISC: Leukocyte images for segmentation and classifcation
BCCD: Blood cell count and detection
Grad-CAM: Gradient-weighted class activation mapping
SVM: Support vector machine
RF: Random forest
CNN: Convolutional neural network
RNN: Recurrent neural network
B: Basophil
M: Monocyte
E: Eosinophil
N: Neutrophil
L: Lymphocyte
FC: Fully-connected
SAM: Spatial attention module
CAM: Channel attention module
OA: Overall accuracy
AP: Average precision
AR: Average recall
AF1: Average F1-score
TP: True positive
FP: False positive
TN: True negative
FN: False negative
CSAM: Channel and spatial attention module
GAP: Global average pooling
GMP: Global max pooling
THA: The highest accuracy
CD: Channel dimension
SD: Spatial dimension
TD: Two dimensions
SE: Squeeze and excitation
ECA: Efficient channel attention
RM: ResNet module
DM: DenseNet module
